# Very Early Onset Psychosis and the Autism Spectrum – Challenges of Differential Diagnosis

**DOI:** 10.1192/j.eurpsy.2025.901

**Published:** 2025-08-26

**Authors:** A. F. Silva, A. C. Rodrigues, A. C. Matias-Martins, F. Santos, P. Coelho, R. M. Lopes, T. Vieira, V. Melo

**Affiliations:** 1 Psiquiatria, Unidade Local de Saúde do Médio Tejo, Tomar, Portugal

## Abstract

**Introduction:**

Schizophrenia (SCZ) and Autism Spectrum Disorder (ASD) are complex neurodevelopmental disorders with overlapping cognitive, social, and behavioral symptoms. Although each has distinct diagnostic criteria, shared traits such as impaired social cognition, communication difficulties, and atypical behaviors, often blur the distinction between them. This overlap is particularly challenging in cases of very early onset psychosis (before age 13), where symptoms like social withdrawal, unusual behaviors, and communication difficulties closely mirror those of ASD, complicating accurate diagnosis.

**Objectives:**

This study aims to explore the diagnostic challenges of distinguishing between ASD and early psychosis through a comprehensive review of published literature and a case report.

**Methods:**

A bibliographic review was conducted using articles from PubMed, focusing on the terms “Autism Spectrum Disorder”, “Early Psychosis”, and “Early Onset Schizophrenia”. Additionally, a case report was presented to illustrate the complexities in differentiating these overlapping conditions.

**Results:**

This study highlights the difficulty of distinguishing ASD from early psychosis due to overlapping symptoms, particularly in young patients. ASD is typically characterized by persistent social communication difficulties and repetitive behaviors, while early psychosis involves hallucinations, delusions, and disorganized thinking. However, some children with ASD may also exhibit psychotic-like symptoms, such as paranoia or unusual perceptual experiences, mimicking early-onset schizophrenia. These findings underscore the importance of comprehensive diagnostic assessments that include developmental history, symptom trajectory, and family background. Increasing evidence shows that ASD and early psychosis share genetic, neurobiological, and environmental risk factors, supporting the idea of a neurodevelopmental continuum where both conditions may be viewed as different points along a shared spectrum of neurodevelopmental disruption.

**Conclusions:**

This work calls for a more integrated approach to diagnosing ASD and early psychosis, especially in cases of very early onset. A continuum model suggests these disorders may represent points along a spectrum of neurodevelopmental disorders rather than entirely separate entities. Future research should prioritize long-term studies to identify specific markers, such as genetic, brain imaging, and cognitive profiles, that can better differentiate between ASD and early psychosis and guide more targeted, personalized interventions.

**Disclosure of Interest:**

None Declared

